# Detection of Foreign Bodies in Soft Foods Employing Tactile Image Sensor

**DOI:** 10.3389/frobt.2021.774080

**Published:** 2021-12-03

**Authors:** Kazuhiro Shimonomura, Tinghsuan Chang, Tomomi Murata

**Affiliations:** Department of Robotics, Ritsumeikan University, Kusatsu, Japan

**Keywords:** food inspection, tactile sensing, camera, image analysis, tactile image sensor

## Abstract

In the inspection work involving foodstuffs in food factories, there are cases where people not only visually inspect foodstuffs, but must also physically touch foodstuffs with their hands to find foreign or undesirable objects mixed in the product. To contribute to the automation of the inspection process, this paper proposes a method for detecting foreign objects in food based on differences in hardness using a camera-based tactile image sensor. Because the foreign objects to be detected are often small, the tactile sensor requires a high spatial resolution. In addition, inspection work in food factories requires a sufficient inspection speed. The proposed cylindrical tactile image sensor meets these requirements because it can efficiently acquire high-resolution tactile images with a camera mounted inside while rolling the cylindrical sensor surface over the target object. By analyzing the images obtained from the tactile image sensor, we detected the presence of foreign objects and their locations. By using a reflective membrane-type sensor surface with high sensitivity, small and hard foreign bodies of sub-millimeter size mixed in with soft food were successfully detected. The effectiveness of the proposed method was confirmed through experiments to detect shell fragments left on the surface of raw shrimp and bones left in fish fillets.

## 1 Introduction

Food safety is a major concern for consumers. Therefore, for companies engaged in food production and processing, quality control of food products is among the most important issues. In food factories, food pretreatment process such as washing, removal of inedible parts, and cutting of food materials takes place. In this process, the detection of foreign bodies can also be an important consideration. The term *foreign bodies* here refers to undesirable solids present in food products, and it includes intrinsic foreign bodies such as shell and bone in animal products, and extrinsic foreign bodies such as stones, strings, and insects. In order to detect foreign bodies in food automatically and efficiently, food inspection machines can be used. Food inspection machines for detecting foreign objects currently include metal detectors, X-ray inspection machines, and image inspection machines. Metal detectors can detect metal contamination by detecting changes in the target’s magnetic field which is affected by metal passing through it (Yamazaki et al., 2002). X-ray inspection machines are used to see through manufactured products (Mohd Khairi et al., 2018). The captured image can be analyzed to distinguish between foreign objects and other objects, and thus X-ray inspection machines can detect a wider range of objects than metal detectors, such as stones, glass, bones, and plastics. Such machines can also check for cracks and chips in weight and appearance (Mohd Khairi et al., 2018). Image inspection machines process camera images to inspect the appearance and size of the food product, and various computer vision algorithms have been developed depending on the target food (Brosnan and Sun, 2004). These conventional inspection methods for foods are noncontact methods that can detect foreign objects. However, when it comes to foreign objects that cannot be easily identified visually or small objects such as small bones, detection can be difficult, and in many cases, the final inspection is performed by human eyes or by touch. For example, if the food is soft and the foreign object to be detected is harder than the food, such as a bone left in a raw fish fillet, it may only be detectable through touch. This entails significant human resources and time, which add cost.

Tactile sensing in humans is distributed or arrayed to obtain the position and shape of the contact area. Tactile sensors with similar performance to a human fingertip should have a spatial resolution of 1–2 mm and encompass 50 to 100 sensing points (Dahiya et al., 2010). Furthermore, human tactile sense can clearly perceive submillimeter unevenness in terms of height. Depending on the type of product, some inspection work relies on the extremely sensitive tactile function of the human hand. To automate such inspection work, a tactile sensor with high spatial resolution and high sensitivity is required.

Tactile sensors that can obtain high spatial resolution and rich information using a camera have been proposed (Shimonomura, 2019). Here, we refer to such sensors as *tactile image sensors*. Various types of tactile image sensors have been developed to date (Shimonomura, 2019), and other new tactile image sensors have been proposed recently (Kuppuswamy et al., 2020; Lambeta et al., 2020). Our research group has developed several tactile image sensors and have tried to use them primarily for grasping control of robot hands and manipulation tasks so far (Shimonomura and Nakashima, 2013; Shimonomura et al., 2016; Nozu and Shimonomura, 2018). Tactile image sensors generally consist of a camera and a tactile skin that converts physical contact into a light signal that can be captured by a camera. Depending on how the contact is converted into optical information, it can be classified into several types (Shimonomura, 2019). For example, the marker displacement method is one of the most popular methods, in which a camera measures the displacement of a visual marker embedded in a soft material (Ward-Cherrier et al., 2018; Sferrazza and D’Andrea, 2019; Hofer et al., 2021). This method is suitable for detecting the magnitude and direction of a force. In the reflective membrane method, the camera captures small deformations on the surface of a sheet of flexible material covered with a reflective membrane (Yuan et al., 2017). Because minute irregularities on the sensor surface are emphasized by the illumination applied from the side, even very small irregularities in the contact area, such as human fingerprints, can be visualized. In this study, we develop a tactile image sensor using the reflective membrane method to detect minute foreign objects.

The main application of tactile image sensors is in robotic manipulation. Therefore, most of the sensors are of the fingertip or gripper type (Yamaguchi and Atkeson, 2019; Wang et al., 2020; She et al., 2021; Song et al., 2019). In addition, this type of sensor has been used for object identification (Li and Adelson, 2013) and texture classification (Patel et al., 2021). In (Lepora and Ward-Cherrier, 2016), a fingertip-shaped tactile image sensor was applied for quality control tasks. That demonstrated that their tactile quality control robot equipped with a tactile fingertip could perform a gap width inspection and identify gaps with submillimeter accuracy.

When using a fingertip-type sensor for inspection, multiple inspection points are touched sequentially one by one while repeatedly moving the sensor up and down. Therefore, when a large number of objects are to be inspected (such as in a food factory), these sensor shapes are not always efficient. Inspection work in food factories generally requires high inspection speed. To meet this requirement, we propose a cylindrical tactile image sensor. The cylindrical sensor surface is rolled over the inspection object to efficiently acquire a high-resolution tactile image. In this paper, we describe a method for detecting small and hard foreign bodies in soft foods using a cylindrical tactile image sensor. In the following sections, we describe the details of the cylindrical tactile image sensor. Then, we consider the conditions for detecting small foreign objects placed on soft objects through experiments using artificial objects. Finally, we apply the sensor to detection of foreign bodies in soft foods such as raw shrimp, raw fish fillet, and minced chicken.

## 2 Materials and Methods

### 2.1 Principle of Detection of Hard Foreign Bodies in Soft Food Using a Tactile Image Sensor

A tactile image sensor using a camera detects the necessary tactile information through the image acquired by the camera observing the deformation of the sensor surface and the associated phenomena that occur when the sensor surface physically contacts an object (Shimonomura, 2019). The shape of the sensor surface is often flat or hemispherical, and the sensor surface is generally fixed to the camera.

In food factories, the target food ingredients are often placed in large quantities on an inspection table or belt conveyor. When using a flat tactile sensor found in many studies on tactile image sensor, the inspection is performed by moving the sensor up and down and touching the objects one by one. This method using a flat tactile sensor is very time consuming. Because inspection work in food factories requires reasonable inspection speed, it is necessary to perform inspections more efficiently. Therefore, in this study, we used a cylindrical-shaped sensor and acquired images by rolling it over objects on an inspection table or belt conveyor. This allows for more efficient inspection because it allows continuous inspection without moving the sensor up and down. We previously presented a prototype of the cylindrical tactile image sensor in (Shimonomura, 2019), and a similar sensor was reported in (Cao et al., 2021), in which the geometry of the cylindrical sensor was discussed.

**FIGURE 1:**
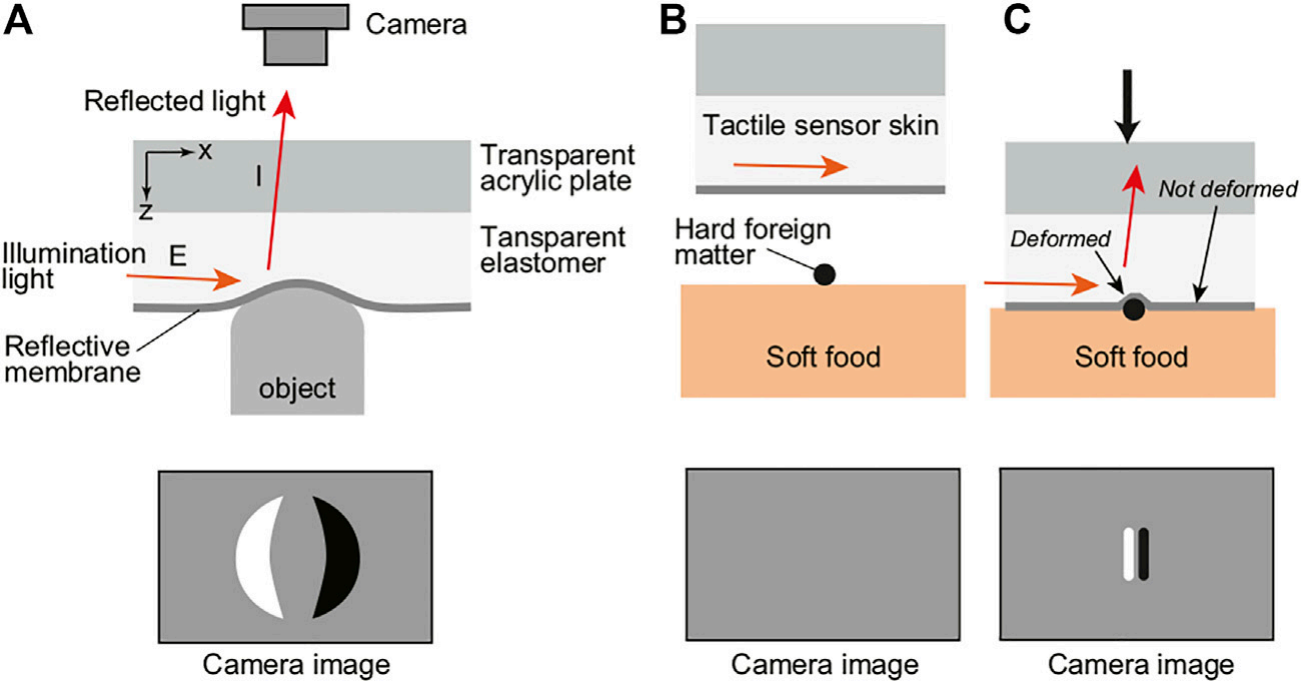
Principle of contact sensing with the tactile image sensor and detection of foreign bodies in soft foods. **(A)** basic structure of the reflective membrane-based tactile image sensor. Because the illumination light was installed only on the left side of the figure, the left side of the deformation which is exposed to the illumination light looks bright on the camera image, and the right side looks dark on the camera image due to the shadow. **(B)** and **(C)** mechanism of detection of foreign bodies. When pressing the soft food by the tactile sensor, the soft food which is softer than the elastomer in the sensor deforms and the sensor skin does not deforms. Since the foreign body is harder than the sensor skin, deformation of the sensor skin occurs at this point, and it can be detected on the camera image.

In the cylindrical tactile image sensor used in this study, the method of conversion from physical contact to light signal is the reflective membrane-based sensor, as used in GelSight (Yuan et al., 2017) and a sensor we previously developed (Nozu and Shimonomura, 2018). Figure 1A shows the basic structure of the reflective membrane-based tactile image sensor used in this study. The surface of the transparent elastomer sheet is coated with a reflective membrane so that the illuminating light from the light source is well reflected. When an object makes contact with the sensor surface, the elastomer surface deforms, and some illumination light is reflected by the reflective membrane at the deformed point. By representing the surface as a height function, *z* = *f* (*x*, *y*), and assuming a diffuse reflective surface, the reflected light intensity *I* (*x*, *y*) can be expressed as follows:

$$I\left(x,y\right)=R\left(\frac{\partial f}{\partial x},\frac{\partial f}{\partial y}\right)E\left(x,y\right)$$ (1)

where *R* is a reflective function defined based on the gradient of the surface *∂f*/*∂x* and *∂f*/*∂y*, and *E* is the illumination light intensity. The intensity of the reflected light can be changed by changing the gradient of the surface. As a result, a shading image reflecting these deformations appears in the image obtained from the camera. Of the deformation of the sensor surface, the side exposed to the illumination light looks bright on the image, and the opposite side looks dark on the image due to the shadow.

In this manner, the sensor responds to the unevenness of the contact surface, and the steeper the change in unevenness, the more clearly the response tends to appear on the image. On the other hand, even if the force applied to the sensor surface is large, if the shape of the sensor surface does not change, there is no response. For example, even if the sensor is firmly pressed to a hard flat surface without unevenness, no change occurs in the image. Figure 1B shows the expected mechanism with respect to the detection of hard foreign bodies on soft food. When pressing the sensor onto soft food, the food itself deforms. If the food is softer than the surface of the sensor, the foodstuff deforms largely and the surface of the sensor will not deform and no response will occur. On the other hand, when there is a hard foreign object, some of the hard foreign object will deform the sensor surface owing to the relationship between the hardness of the sensor surface and the hardness of the food. This allows the sensor to respond to and detect foreign objects.

### 2.2 Cylindrical Tactile Image Sensor

**FIGURE 2:**
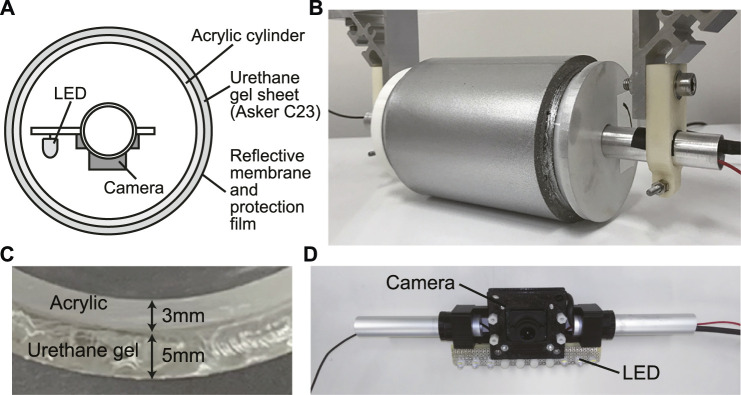
The cylindrical tactile image sensor. **(A)** internal structure of the sensor seen from the side, **(B)** picture of the sensor, **(C)** enlarged view of the sensor skin. Outside surface of the urethane gel layer is coated by reflective membrane. **(D)** camera and illumination set in the cylindrical sensor body.

Figure 2A shows the structure of the cylindrical tactile image sensor used in this study. A layer of soft sensor surface is formed on the outer surface of a transparent hard acrylic cylinder. A transparent urethane gel sheet was used for this purpose. A reflective membrane (silver coating) is then formed on the surface of the sensor surface. To protect the surface, a thin sheet of polyurethane was used to cover the reflective surface. Using this structure, the entire outer surface of the cylinder becomes the contact detection surface. A camera is placed inside the cylinder. The camera is fixed to a shaft that does not rotate with the cylinder, and photographs the contact area from inside the cylinder. LEDs for illumination are placed near the camera to illuminate the sensor surface from within. Figure 2B shows a picture of the cylindrical tactile image sensor. The diameter and width of the acrylic pipe used for the cylindrical sensor body were 80 and 120 mm, respectively. The elastomer sheet used in the sensor surface was an urethane gel sheet (EXSEAL Co., Ltd.) with a thickness of 5 mm and with Asker C23 hardness. The outer surface of the urethane gel sheet was coated with silver paint and then covered with a 20 *μ*m thick film of polyurethane (3M Tegaderm Roll). A camera (IDS UI-3251LE-C-HQ) and a white LED illumination light source were placed inside the cylinder. The resolution of the camera is 1,600 × 1,200 pixels. The cables for the camera and illumination light source were routed through the hollow shaft and were not affected by the rolling motion of the sensor body (Figure 2D).

**FIGURE 3:**
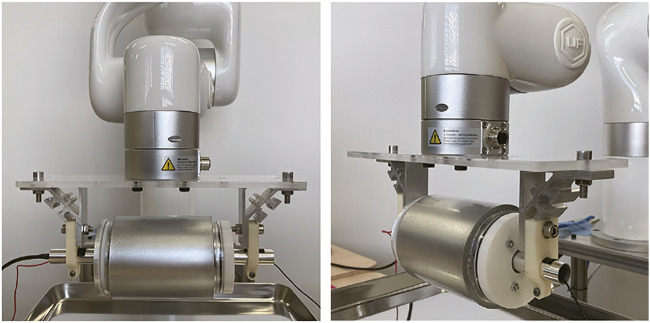
Pictures of the robotic arm equipped with the cylindrical tactile image sensor.

In actual use in a food factory, the cylindrical tactile image sensor can be fixed over a belt conveyor, assuming that the foodstuff is being moved by a belt conveyor. However, for the laboratory experiments in this study, the sensor was attached to a robotic arm (UFACTORY xArm 5 Lite) so that it could move at a certain speed in a specific direction (Figure 3).

### 2.3 Tactile Image Processing

**FIGURE 4:**
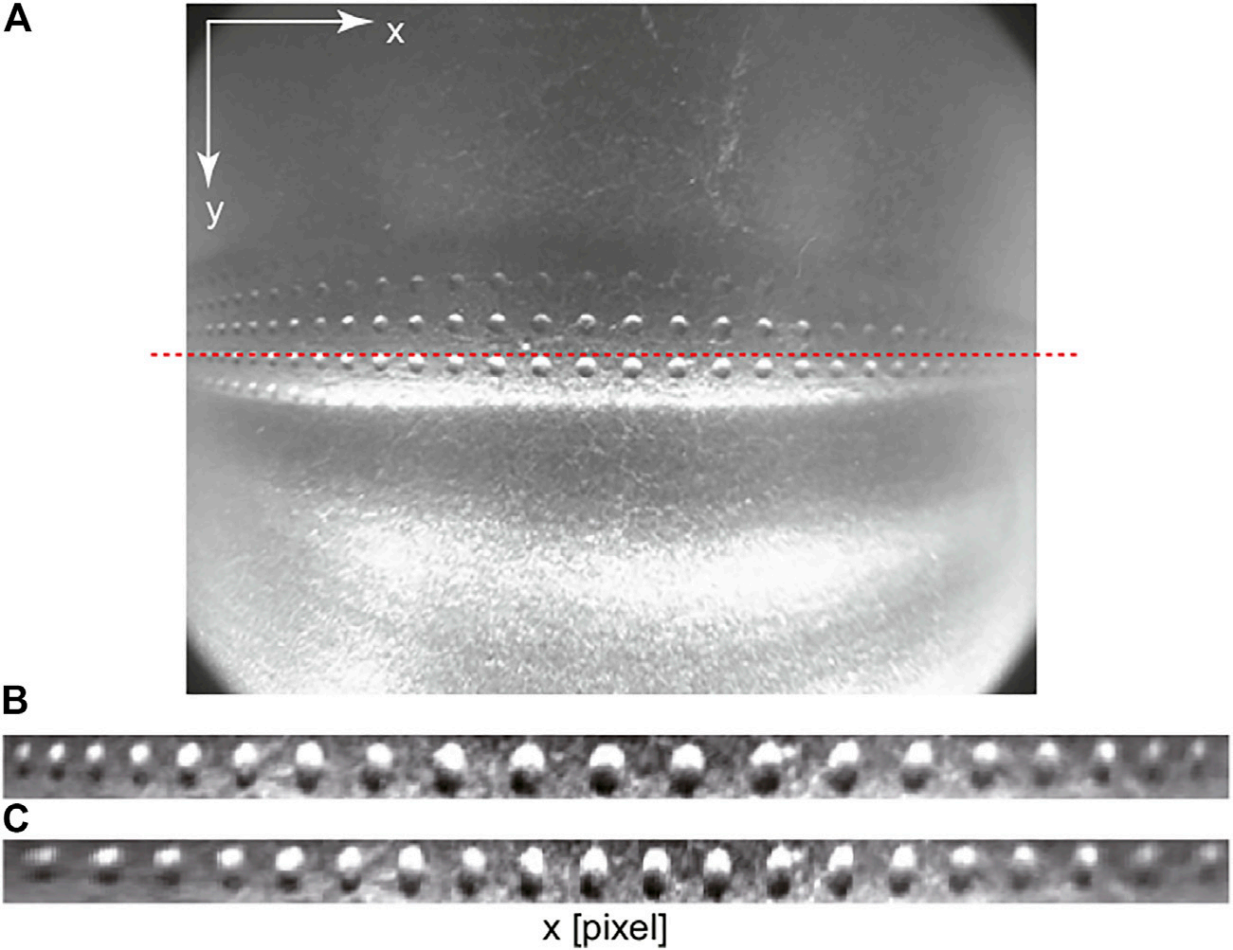
Output image obtained from the tactile image sensor. **(A)** raw image. Red dashed line shows the center line in the *y* coordinate. **(B)** extracted image from the contact region without calibration, **(C)** calibrated image.

Figure 4 shows a camera image obtained from the cylindrical tactile image sensor. The image was captured when the sensor was in contact with a board with periodically arranged holes. In the image, a response can be seen in the row near the middle of the image, indicating that this area is in contact with the board. In this study, the line near the middle (shown by the red dashed line in Figure 4) that makes contact when the sensor is pressed against the object from above is extracted and used. Because a short focal length lens is used, a large lens distortion is observed. To determine where the foreign object is detected, it is necessary to calibrate for the lens distortion to match the image coordinates to the external coordinates in addition to the position of the sensor itself. To calibrate the lens distortion, a standard camera calibration tool, such as those implemented in OpenCV (https://opencv.org/) which is an open source library for computer vision, can be used before the camera is placed inside the cylindrical sensor body. However, even after the camera is placed inside the cylinder of the tactile sensor, the *x*-direction of the image can be calibrated by a simpler method, that is, polynomial fitting can be applied by using the sensor output image responding to a flat plate with equally spaced bumps and holes to estimate the lens distortion in the *x*-direction. Figure 4B shows the image area of the row near the middle extracted from the original image shown in Figure 4A, and Figure 4C shows the image with correction in the *x*-direction after polynomial fitting.

**FIGURE 5:**
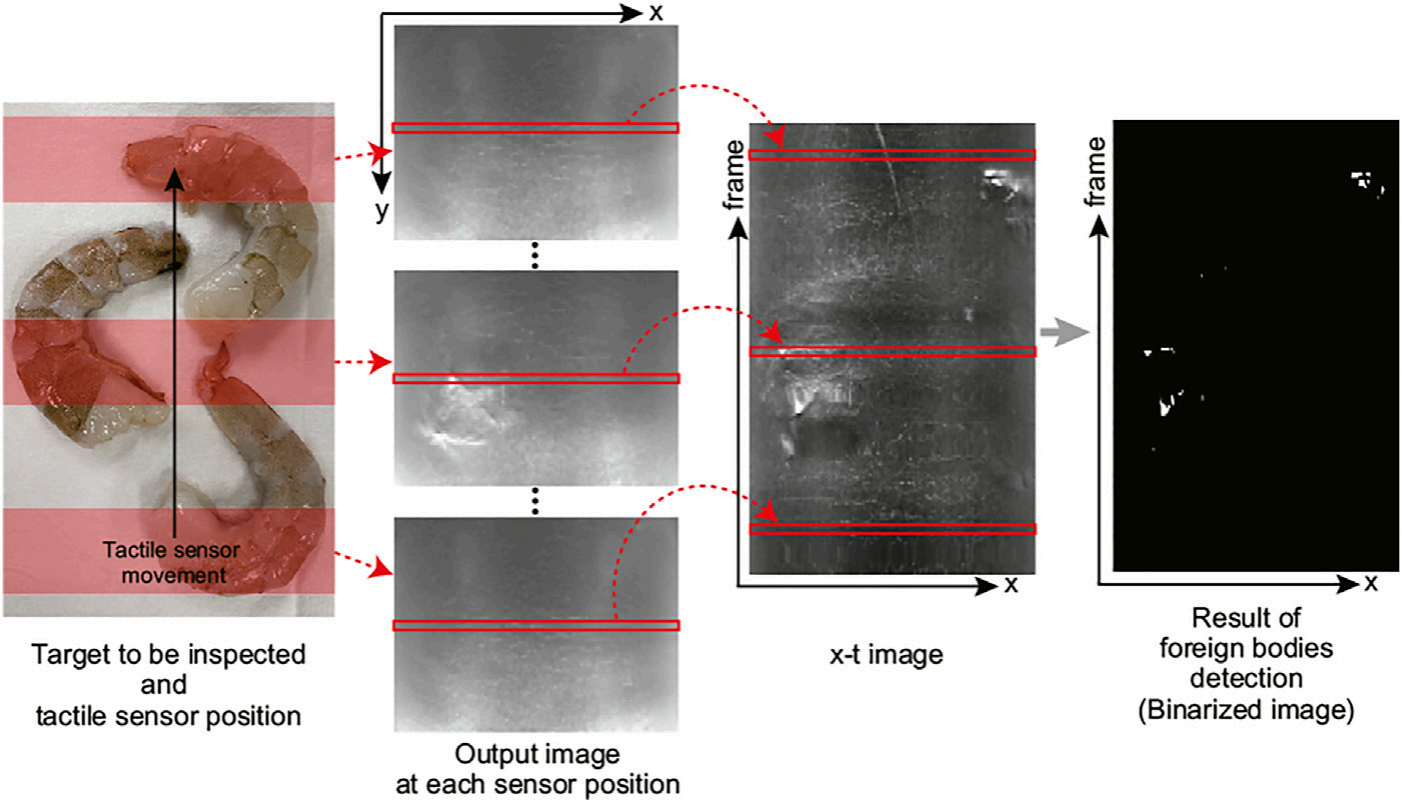
Tactile image processing for detecting foreign bodies in the proposed cylindrical tactile image sensor.

Figure 5 shows the flow of the tactile image processing to detect foreign bodies when continuously inspecting a target area. The cylindrical tactile sensor acquires image sequence while rolling over the target. The speed of the image acquisition depends on the frame rate of the camera. Then, a line around the middle (that corresponds to the location where makes contact) is extracted from each frame, and these lines are connected in the order of the frames to create an *x* − *t* image, of which size is 1,600 × (frame number). As mentioned previously, the deformation on the sensor surface occurred by the small hard foreign bodies in soft food appears as bright and dark pixel in the camera image. Here, the foreign bodies is detected by detecting bright pixel through simple binarization using fixed threshold value.

## 3 Results

### 3.1 Detection of Hard Small Object in a Soft Object

**FIGURE 6:**
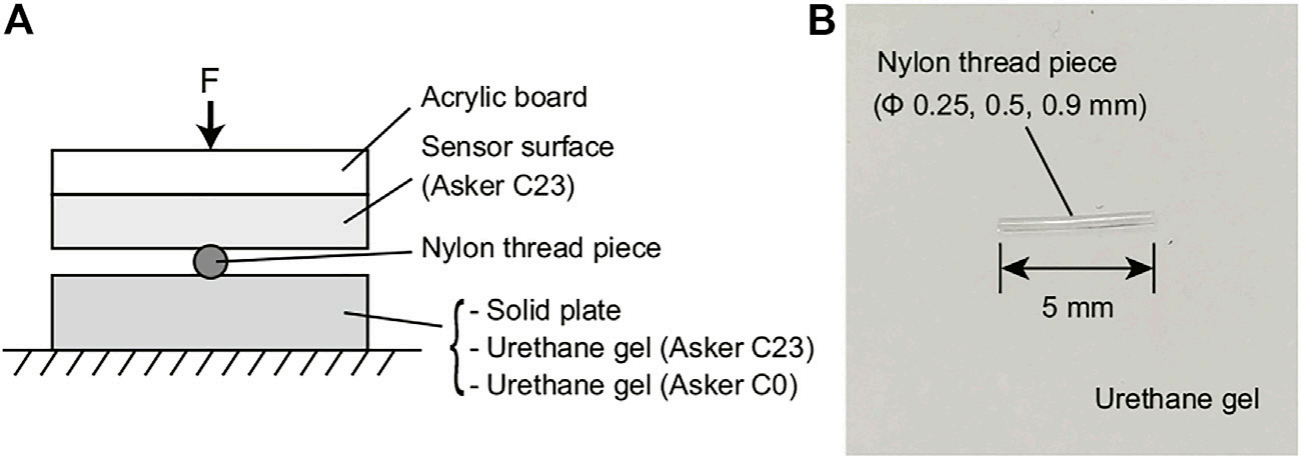
Experimental setup for hard object piece detection experiment using artificial objects. **(A)** arrangement of the materials. Three kinds of object ware used; Solid plate (metal tray), urethane gel with Asker C23 hardness (EXSEAL Gel tack series), and urethane gel with Asker C0 hardness (EXSEAL seitai gel series). On these object, the nylon thread piece with 5 (mm) of length and with three different diameter were arranged; *ϕ*0.25 (mm), *ϕ*0.5 (mm), and *ϕ*0.9 (mm). **(B)** picture of the nylon thread piece located on the urethane gel sheet.

Prior to the experiments using real food, we conducted preliminary experiments using artificial objects to clarify the conditions under which a hard foreign body can be detected in soft foodstuff. Figure 6 shows the experimental setup. A sheet of soft urethane gel sheet was used instead of soft food. Two levels of hardness were used: Asker C23 and Asker C0. The Asker C23 urethane gel is the same material used for the tactile sensor skin. For comparison, an experiment was conducted on a solid plate (metal tray) without a soft material. Nylon threads whose length was 5 mm, having diameters of 0.25, 0.5, and 0.9 mm were used as small hard foreign objects. The nylon thread pieces were placed on top of the urethane gel or solid plate, and the sensor output image was acquired by pressing the tactile image sensor to the thread. Figure 6B shows a picture of the nylon thread placed on white urethane gel.

**FIGURE 7:**
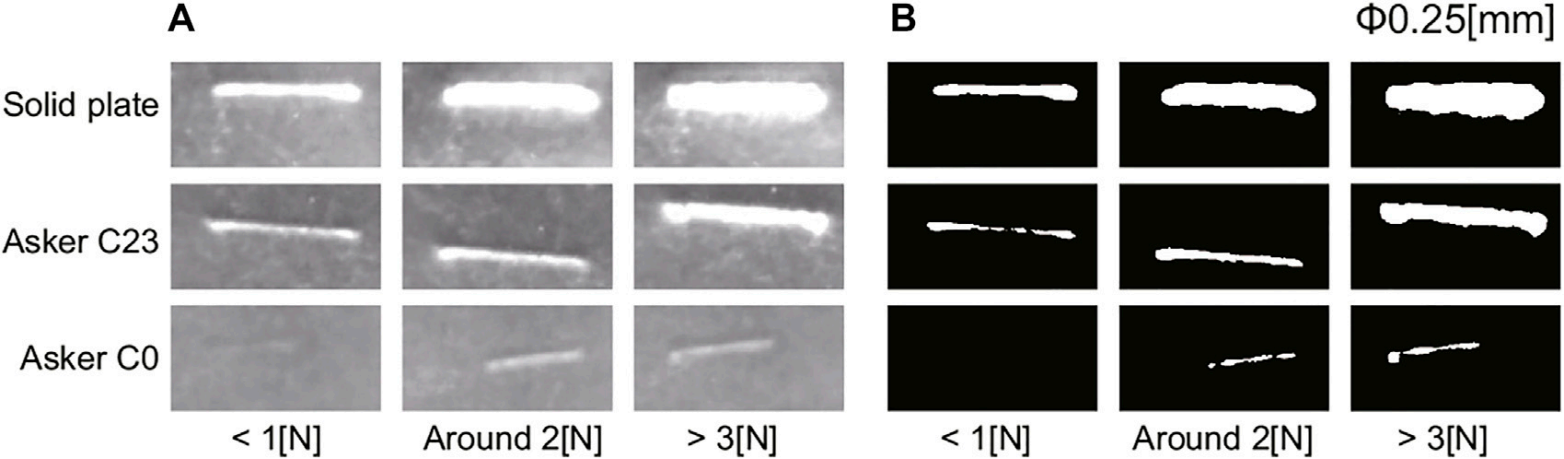
Experimental results of hard object piece detection: Output image for *ϕ*0.25 (mm) nylon thread piece. **(A)** raw image from the sensor, **(B)** binarized image. The area shown by white pixel is used as response of the sensor for each condition.

Figure 7 shows some output images obtained from the tactile image sensor for a 0.25 mm diameter nylon thread. In each image, a 100 × 300 pixel region around the response to the nylon thread was cropped from the output image. From the top row down, each row shows the tactile sensor output images for a solid plate, a urethane gel with hardness Asker C23, and a softer urethane gel with hardness Asker C0, respectively. The image for the Asker C0 material exhibited a weak response, but that for the solid plate exhibited a very strong response. The harder the material underneath the nylon thread, the larger the response, because the nylon thread presses more deeply into the sensor surface, causing greater deformation of the sensor surface. From left to right columns, the images were taken when the pressing force on the sensor was less than 1 N, approximately 2 N, and 3 N or higher, respectively. The larger the pressing force, the larger the response. When the soft material is Asker C0 and the pressing force is approximately 1 N, there is almost no response. On the other hand, when the object under the thread is a soft material with Asker C23 hardness or a solid plate, a clear response is obtained even when the pressing force is low, less than 1 N.

**FIGURE 8:**
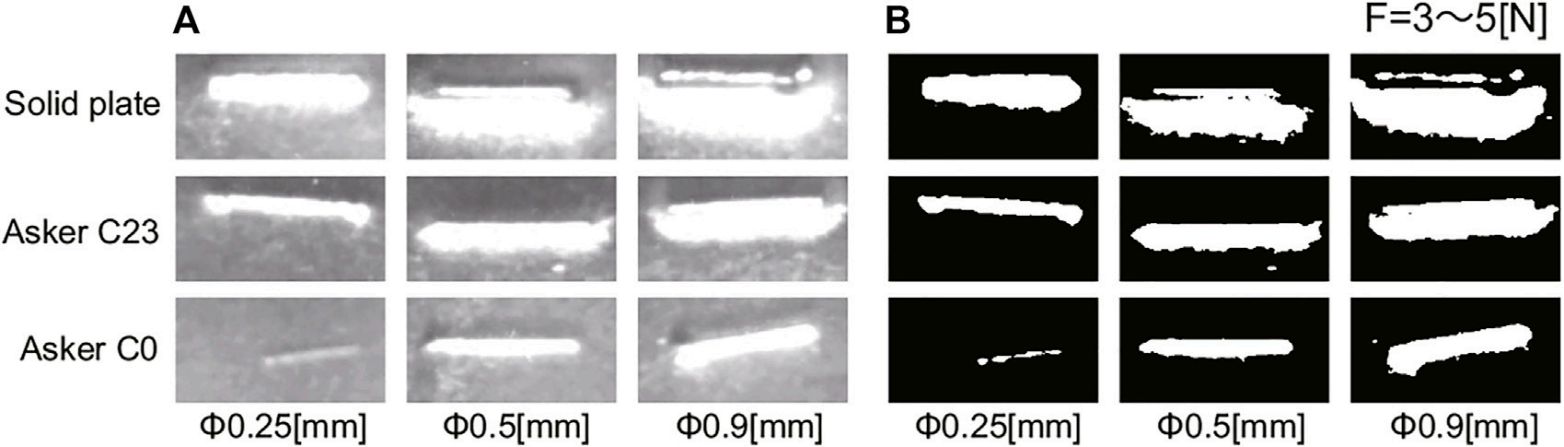
Experimental results of hard object piece detection: Output image for 3 to 5 (N) of pushing force. **(A)** raw image from the sensor, **(B)** binarized image.

Figure 8 shows some of the output images of the tactile image sensor when the pressing force of the sensor was between 3 and 5 N. As indicated by Figure 7, the harder the object under the nylon thread is, the larger the response. Starting from the left column, the images were taken when the diameter of the nylon thread was 0.25, 0.5 and 0.9 mm, respectively. The larger the diameter of the nylon thread piece is, the larger the response. This is because the larger the size of the hard foreign object, even on soft material, the more it bites into the sensor surface, causing greater deformation of the sensor surface.

Figure 7B and Figure 8B show the binarized images obtained from the output image of the tactile image sensor shown in Figure 7A, Figure 8A, respectively. Simple binarization with a fixed threshold value successfully extracts the response to the nylon thread. The area of the response region, represented by white pixels in the binarized image, was used to evaluate the strength of the response in each condition.

**FIGURE 9:**
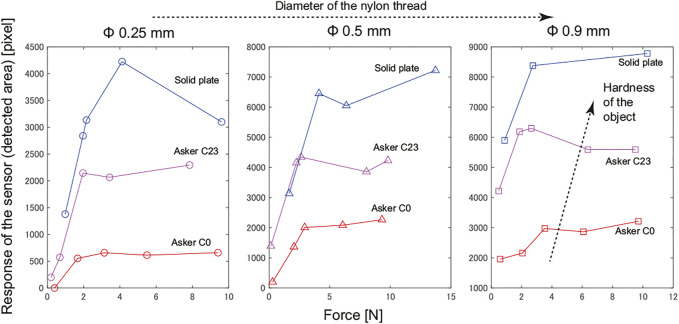
Experimental results of hard object piece detection: Relationship between sensor response (detected area) and pushing force in various conditions.

Figure 9 summarizes the results obtained in this experiment using artificial objects. In all graphs, the vertical axis indicates the area of the foreign object in the binarized image, and the horizontal axis represents the pressing force of the tactile image sensor. The three graphs show the results for different diameter of the nylon thread, and in each graph, the results for different hardness of the material on which the nylon thread piece was placed are compared. Although there were some exceptions, in all graphs, the response tended to increase as the pressing force increased, and the response appeared to become saturated when the pressing force exceeds 4 N. The harder the material placed under the nylon thread piece is, the larger the response. By comparing the plots represented by the same color in these three graphs, we can see that the larger the diameter of the nylon thread, the larger the response.

These results demonstrates that even in the condition with the smallest response, which is the combination of the 0.25 mm diameter nylon thread and Asker C0 urethane gel, if the tactile image sensor is pressed with a force of 4 N or higher, the response will be sufficiently large to allow detection. A clearer response was obtained for thicker nylon thread and harder object even for lower pressing force values.

### 3.2 Experiments Using Food

**FIGURE 10:**
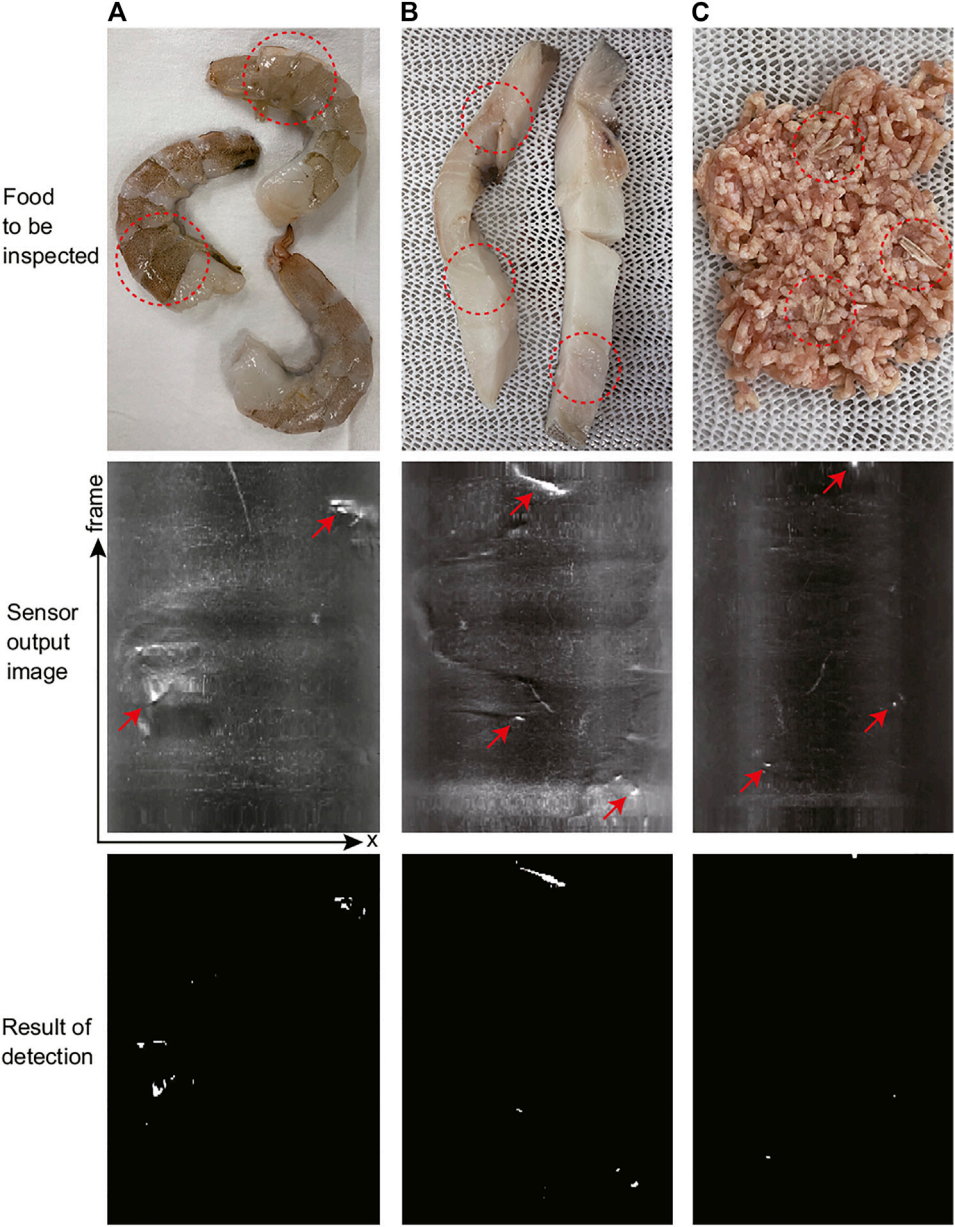
Experimental results for foods. **(A)** raw shrimp, **(B)** raw fish fillet, and **(C)** minced chicken. From top to down, photo of the real foods, raw output images from the tactile image sensor (*x* − *t* image obtained by stacking single line extracted from each frame), and results of foreign bodies detection (binarized image).

We conducted experiments to detect hard foreign bodies using actual food. The foods used were raw shrimp (Figure 10A), raw fish fillets (Figure 10B), and minced chicken (Figure 10C). In food factories, shrimp is often used as an ingredient in ready-to-eat foods. The hard shrimp shells should be removed, but some shell material may remain. Filleted fish should be deboned before packaging, but some small bones may be left behind. In these cases, workers sometimes need to find any remaining shells and bones by touching the product in food factories. In addition, minced meat may have small bone fragments mixed in during processing, and it is desirable to detect and remove them. In all cases, hard foreign bodies are mixed into soft food, and we attempt to detect the foreign bodies using the proposed cylindrical tactile image sensor.

In Figure 10, although somewhat difficult to see foreign bodies in food, there are small hard foreign bodies present in the area circled by red dashed line. In the experiment using raw shrimps, two out of the three shrimp have some shell pieces remaining and for comparison, one shrimp had its shell completely removed. In the fish fillet, there are bones present in three locations. You cannot see these bones in the figure, but when you touch these locations of the fish fillet used in the experiment, you can feel the hard bones. For the minced chicken, three small pieces of chicken bones were added to the minced meat prior to the experiment.

We acquired output images by rolling the cylindrical tactile image sensor over these food materials at a constant speed of approximately 30 mm/s. The middle row of Figure 10 shows the images acquired for each type of food. From each frame of the sensor output image, the lines corresponding to the contact region were extracted and concatenated in frame order to form an *x* − *t* space image, as mentioned in Section 2.3. For each food, it took approximately 10 s to scan the food item from the bottom to the top of the image, and the vertical axis of the *x* − *t* image corresponds to this timing. The brighter the pixels, the larger the response; furthermore, the largest response occurs where the hard foreign bodies are located in the food, as indicated by the red arrows. On the other hand, there is little response to the soft foods themselves. The binarized images are shown in the bottom row. In each food sample, all hard foreign bodies were detected, as expected. There were almost no false positives in areas where there were no foreign bodies, but one false positive was observed in the raw shrimp results, slightly above the center of the image.

## 4 Discussion

### 4.1 Mechanism of Hard Piece Detection in Soft Object

**FIGURE 11:**
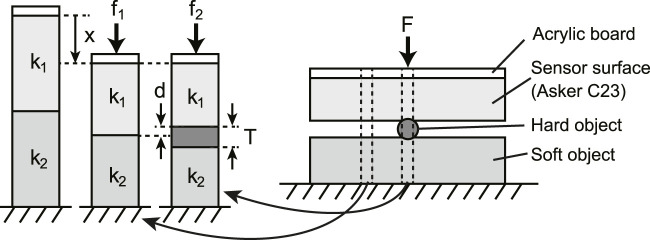
Simple spring model of foreign bodies detection mechanism with the tactile image sensor. Right image shows the same situation as Figure 6A, where the sensor skin is pushing the hard foreign object located on the soft food. Considering a small compartment extracted from two different part of the right figure, the part including hard object and the part without hard object, to estimate the static characteristics of the deformation of sensor skin and soft object in these two compartments.

The experimental results indicated that the larger the diameter of the nylon thread and the harder the object placed under the nylon thread, the greater the response of the tactile image sensor. This result seems intuitively reasonable, but we discuss the mechanism further based on the model shown in Figure 11. Consider the case where a hard nylon thread is placed on top of a soft object and the sensor surface is pressed against it. We consider the area where the nylon thread is inserted and the area where the sensor surface is in direct contact with the soft object as two separate compartments. In each compartment, the flexible material layer that makes up the sensor surface and the soft object layer are considered as springs with spring constants *k*_1_ and *k*_2_, respectively. We consider a state in which a force is applied from the natural length state (where no force is applied from above) and a state in which a force is applied and a displacement *x* occurs.

For the model of the area where the sensor surface and the soft object are in direct contact, if the displacements of the flexible material layer that makes up the sensor surface and the soft object layer are *x*_1_ and *x*_2_, respectively, then *x* = *x*_1_ + *x*_2_, and the ratio of each displacement is the reciprocal of the ratio of the spring constants. Therefore, the ratio with respect to the total displacement is

$$\frac{x_{1}}{x_{2}}=\frac{k_{2}}{k_{1}}$$ (2)

For the model where the sensor surface and the soft object sandwich a piece of nylon thread, let the height of the nylon thread be *T*. Because of the hard acrylic plate on the sensor surface, the overall displacement is *x* as in the above case. However, assuming that the nylon filament is sufficiently hard so that it does not deform, the displacement between the sensor surface and the soft object layer will be *x* + *T*. Assuming a linear spring, for the displacement corresponding to *T*, the ratio of the displacement between the flexible material layer that makes up the sensor surface and the soft object layer will be the reciprocal of the ratio of the spring constants. Based on the principle shown in Figure 1, the magnitude of the tactile sensor response depends on the difference *d* in the sensor surface position between the areas without and with the hard nylon filament. Therefore, *d* is the displacement of the flexible material layer that makes up the sensor surface for the amount corresponding to *T*

$$d=\frac{k_{2}}{k_{1}+k_{2}}T$$ (3)

In the compartment of the model, if the natural length and cross-sectional area of the flexible material layer that makes up the sensor surface and the layer of the soft object are the same, the spring constant can be replaced by the Young’s modulus of the material. The Young’s modulus of the flexible material used was provided by the manufacturer. Using these values, *d* = 0.41*T* if the object is Asker-C0 and *d* = 0.5*T* if the object is Asker-C23. These results indicated that the larger in diameter the nylon thread is, the stronger the sensor response will be because *T* (the nylon thread diameter) is larger. The softer the object is, the smaller the deformation of the sensor surface at the location where the nylon filament is pressed, and thus the smaller the sensor response. However, the quantitative relationship between the amount of deformation of the sensor surface and the strength of the sensor response is not clear; therefore, building a model that can quantitatively explain the sensor response is a future challenge.

### 4.2 Tactile Sensing for Food Inspection

In our experiments, we were able to detect hard foreign objects in three types of soft food. In all cases, the tactile image sensor detected foreign objects that were difficult to detect visually and could require tactile detection (by a human). In this paper, we demonstrated that the proposed sensor works well in some food samples. As a future work, it is necessary to evaluate the detection performance using a large number of target food in the environment of an actual food factory. In the experiment to detect remaining shells in raw shrimp, shells attached to the front side where the sensor surface made contact were detected. However, if a shell is located on the back side of the shrimp, it cannot be detected by touching it from the front side, so the shrimp must be turned to its other side to achieve full inspection. In this case, a mechanism to flip the shrimp over can be added, or a method to inspect the shrimp by sandwiching it between two roller-type sensors can be considered. For practical use, it is necessary to consider installing sensors according to the actual requirements in food factories. It is also necessary to consider how to remove detected foreign matter. The location of the detected foreign object can be determined from its position in the image, and this information can be used for removal purpose.

In this experiment, we acquired images while moving the sensor horizontally at a constant speed. Some of the foodstuffs used were moist, and the magnitude of friction varied depending on the foodstuff. Therefore, in some cases, the roller-type sensor rolled, and in other cases, it slipped over the food surfaces. However, in either case, the process of concatenating partial images from each frame of the output image was not affected. The rotating cylindrical structure of this sensor not only allows continuous inspection over a wide area, but also helps to limit the force applied to the foodstuff in the shear direction.

The force applied to the foodstuff varied depending on the height of the foodstuff and ranged from 2 to 7 N. This was the condition under which a sufficiently large sensor response to foreign matter could be expected based on the results of preliminary experiments. On the other hand, from the viewpoint of keeping the inspection conditions constant, it would be desirable to apply a constant pressing force to food under inspection. Adding such a mechanism to the sensor section is a topic for future work.

### 4.3 Speed of Inspection With Cylindrical Sensor

In the pretreatment of foodstuffs in a food factory, it is generally necessary to process a large amount of foodstuffs, and therefore, inspections should be performed as quickly as possible. In this study, we proposed a cylindrical tactile image sensor that can continuously inspect food while rolling over the food to be inspected, assuming that the food is moving on a belt conveyor. This allows the inspection to proceed smoothly, and the inspection speed was approximately 30 mm/s in the experiments using actual food. The inspection speed should be further increased for practical applications.

In the cylindrical tactile image sensor, the physical length of the contact surface corresponding to the width of one pixel was approximately 100 *μ*m. If only one line of the sensor output image is cut out and the camera is set to a frame rate of 30 fps, the possible scanning speed to avoid missing foreign objects would be 100 *μ*m/(1/30) s = 3 mm/s. If the scanning speed is faster than this, either foreign objects will be missed or the area of the foreign object detected will be small. In this experiment, the area of foreign matter detected appears to be slightly smaller owing to this effect. If a high-speed camera with a frame rate 10 times faster is used, the scanning speed can also be increased by 10 times.

In addition, the scanning speed can be improved by increasing the range of the image to be extracted and used for foreign object detection. If 100 lines are used in the sensor output image, instead of only the center row (one line) on the image corresponding to the lowest point of the cylindrical sensor surface, a foreign object can be detected if it falls within the corresponding 10 mm range in each frame. Therefore, the scanning speed can be increased by a factor of 100, which reaches 300 mm/s and is suitable for the belt conveyor inspection. To adopt this method, it will be necessary to evaluate the detection accuracy when a foreign object touches a position deviating from the lowest point of the cylindrical sensor surface, and to determine the appropriate pressing force so that the contact range between the sensor surface and the food is widened and the detectable range of foreign objects is increased. Such improvements involving higher speeds are future tasks.

## Data Availability

The raw data supporting the conclusion of this article are available from the corresponding author upon request.
